# Cleanliness and Health

**Published:** 1898-09

**Authors:** 


### Cleanliness and Health.

Cleanliness covers the whole field of sanitary labor. Cleanliness means purity of both air and water; cleanliness in and around the house; cleanliness of person; cleanliness of dress; cleanliness of food and feeding; cleanliness in work; cleanliness in the habits of the individual man and woman; cleanliness of life and conversation; purity of life, temperance—all these are directly in man's power.—*Sir B. W. Richardson.*

